# Reactivation of latent HIV-1 by the glucocorticoid receptor modulator AZD9567

**DOI:** 10.1128/jvi.01886-24

**Published:** 2025-01-16

**Authors:** Sharmeen Fayyaz, Rishikesh Lotke, Isabell Haußmann, Moritz Petersen, Eva Müller, Hannah S. Schwarzer-Sperber, Roland Schwarzer, Daniel Sauter

**Affiliations:** 1 Institute for Medical Virology and Epidemiology of Viral Diseases, University Hospital Tübingen 27203 , Tübingen, Germany; 2 National Institute of Virology, Dr. Panjwani Center for Molecular Medicine and Drug Research, International Center for Chemical and Biological Sciences, University of Karachi 63597 , Karachi, Pakistan; 3 Institute for the Research on HIV and AIDS-associated Diseases, University Hospital Essen 39081 , Essen, Germany; Icahn School of Medicine at Mount Sinai, New York, New York, USA

**Keywords:** HIV, latency, reactivation, glucocorticoid receptor, AP-1, LTR

## Abstract

One key determinant of HIV-1 latency reversal is the activation of the viral long terminal repeat (LTR) by cellular transcription factors such as NF-κB and AP-1. Interestingly, the activity of these two transcription factors can be modulated by glucocorticoid receptors (GRs). Furthermore, the HIV-1 genome contains multiple binding sites for GRs. We therefore hypothesized that glucocorticoids and other GR modulators may influence HIV-1 latency and reactivation. To investigate how GR signaling affects latent HIV-1 reservoirs, we assembled a representative panel of GR modulators including natural steroidal agonists, selective and non-selective GR modulators, and clinically approved GR-modulating drugs. The effects of these compounds on HIV-1 reactivation were assessed using latently HIV-1-infected cell lines and primary cells, as well as reporter assays that monitored GR and LTR activities. We found that AZD9567 (Mizacorat), a non-steroidal partial GR agonist, reactivates latent HIV-1 in both lymphoid and myeloid cell lines and primary CD4+ T cells. Conversely, the GR antagonist mifepristone suppresses HIV-1 LTR-driven gene expression. Mechanistic analyses revealed that AZD9567-mediated reactivation partially depends on both GR and AP-1 binding sites in the LTR. In summary, we, here, identify the GR modulator AZD9567 as novel latency-reversing agent that activates LTR-driven gene expression, which may aid in advancing current shock-and-kill approaches in the treatment of HIV-1 infection.

**IMPORTANCE:**

Latently infected cells of people living with HIV are constantly exposed to fluctuating levels of glucocorticoid hormones such as cortisol. In addition, many HIV-infected individuals regularly take corticosteroids as anti-inflammatory drugs. Although corticosteroids are known to affect the activity of the viral long terminal repeat (LTR) promoter and influence ongoing HIV-1 replication, relatively little is known about the effect of corticosteroid hormones and other glucocorticoid receptor (GR) modulators on latent HIV-1. By systematically comparing natural and synthetic GR modulators, we, here, identify a first first-in-class, oral, partial GR agonist that reactivates latent HIV-1 from different cell types. This drug, AZD9567, was previously tested in clinical trials for rheumatoid arthritis. Mutational analyses shed light on the underlying mode of action and revealed transcription factor binding sites in the HIV-1 LTR that determine responsiveness to AZD9567.

## INTRODUCTION

**Fig 1:**
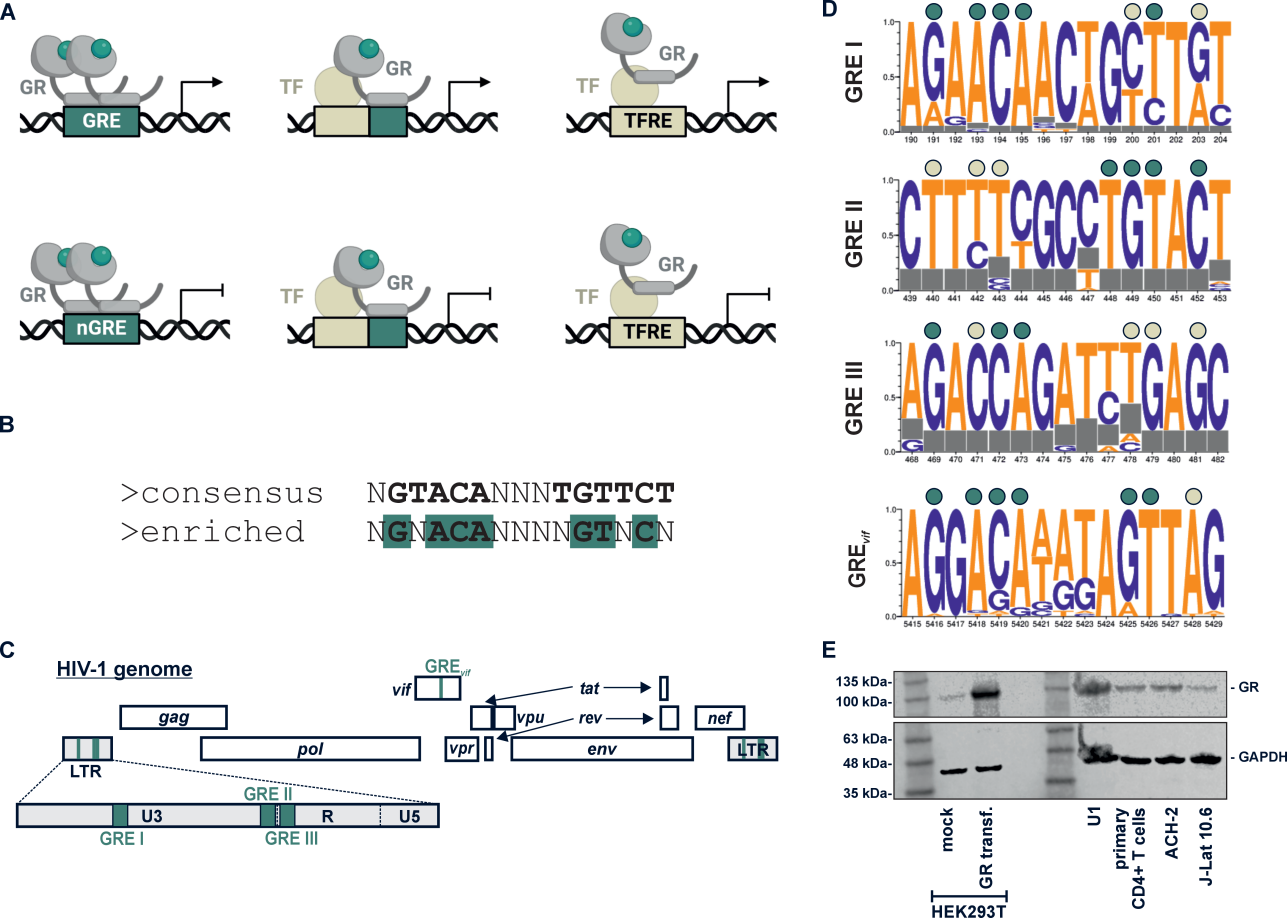
Glucocorticoid response elements in the HIV-1 genome and glucocorticoid receptor expression in HIV-1 target cells. (**A**) GRs (gray) may activate (top) or repress (bottom) transcription by directly binding to GRE in the DNA and/or by interfering with other transcription factors (TF, beige). (**B**) 15 bp core binding sequence of GR homodimers. The canonical consensus binding site is shown on top. Nucleotides that are enriched at experimentally determined GR binding sites are highlighted in green at the bottom. (**C**) Positions of GREs in the HIV-1 genome are indicated in green. (**D**) Sequence logo plots illustrating the conservation of GRE I–III and GRE_vif_ in the genomes of the HIV-1 subtype reference sequences (four representative sequences for each subtype). Green circles indicate the presence of nucleotides enriched in GREs. Beige circles indicate nucleotides that do not match the canonical GR binding site (see panel **B**). (**E**) Endogenous GR protein levels in U1, ACH-2, J-Lat 10.6, HEK293T, and primary CD4+ cells were analyzed by Western blotting. CD4+ cells were activated with phytohemagglutinin. HEK293T cells transfected with a GR expression plasmid served as positive control.

Glucocorticoid receptors (GRs) are expressed essentially in all cells of the human body and mediate the action of glucocorticoid hormones such as cortisol. As such, GRs play a key role in suppressing pro-inflammatory immune responses, controlling glucose metabolism and regulating developmental processes. Like other members of the family of steroid hormone receptors, GRs usually reside in the cytoplasm in the absence of any stimulation. Upon glucocorticoid binding, they translocate into the nucleus where they can act as transcription factors and either activate or suppress gene expression. In a process termed transactivation, GR homodimers directly bind to specific DNA sequences, so-called glucocorticoid response elements (GREs), thereby inducing gene expression (Fig. 1A) (1). However, GRs can also suppress expression of certain target genes by binding to negative glucocorticoid response elements (2). Furthermore, monomeric GR can bind to composite sequence motifs and regulate transcription by cooperating with other transcription factors (1). Finally, GRs can exert their regulatory activity indirectly, without binding to DNA. For example, GR has been shown to suppress gene expression by complexing with and/or sequestering NF-κB or AP-1 (trans-repression) (3, 4).

GREs are frequently described to comprise the 15 bp core sequence N**GTACA**NNN**TGTTCT**, including two hexarepeat half-sites (5) (Fig. 1B). Many GR binding sites, however, are only imperfect palindromes and do not match this consensus sequence. Consistent with this, an earlier study comparing experimentally determined GR binding sites found an enrichment of the following core motif: N**G**N**ACA**NNNN**GT**N**C**N (6). Notably, imperfect second half-sites may also be compensated by nucleotides flanking the 15 bp binding site (7).

GREs are not only present in cellular DNA, but can also be found in viral genomes. For example, four (putative) GR bindings sites have been identified within the HIV-1 genome (8, 9). Three of them (GRE I–III) are located in the U3 and R regions of the viral long terminal repeat (LTR) (Fig. 1C). The fourth one (GRE_vif_) is located within the accessory gene vif. While these four GREs are not perfectly palindromic, sequence logo plots of the HIV-1 subtype reference sequences revealed that the key residues N**G**N**ACA** are largely conserved in the first half-site of GRE I, GRE_vif_, and, in reverse orientation, also in GRE II (Fig. 1D). Moreover, the HIV-1 LTR also harbors several well-characterized bindings sites for the GR targets NF-κB or AP-1, which are important positive regulators of viral gene expression and determinants of viral latency/reactivation (10).

Not surprisingly, the viral LTR promoter has been shown to be responsive to glucocorticoids and other GR-modulating compounds. For example, Mitra et al. demonstrated that dexamethasone, an artificial glucocorticoid, inhibits LTR-driven gene expression in the presence of the viral trans-activator Tat in SK23 T cells, but not in monocytic U937 cells (9). This inhibitory activity depended on an intact GRE III site, but not GRE I or II (9). In contrast, Russo and colleagues observed a suppression of Tat-mediated LTR activation by hydrocortisone and dexamethasone in T lymphocytes (CEM-T4) and monocytes (U937) (11). In agreement with an inhibition of LTR activity by glucocorticoids, dexamethasone suppressed viral replication in primary monocyte-derived macrophages (12), tonsillar tissue explants (13), and microglial cells (14). In stark contrast, however, Soudeyns observed enhanced reverse transcriptase activity of HIV-1-infected U937 and H9 cells upon addition of dexamethasone or cortisol (8). In line with their findings, the GR antagonist mifepristone (=RU-486) suppressed HIV-1 replication in peripheral blood mononuclear cells, and Vpr-mediated reactivation of HIV-1 in latently infected OM-10.1 and U1 cells (15). Similarly, mifepristone inhibited HIV-1 replication in chronically infected ACH-2 cells (16). Some of these seemingly discrepant results may be explained by differences in the dosage of the GR-modulating drugs since trans-repressive but not trans-activating activities of GR are already apparent at lower concentrations of a GR agonist (17). Furthermore, there may be cell line-specific effects, and differences in GR responsiveness may be explained by the presence or absence of viral co-activators such as Tat or Vpr. The latter directly interacts with the glucocorticoid receptor (18) and potentiates its activity (19), further pointing toward an important role of GRs in HIV-1 replication.

Although GRs may modulate HIV-1 LTR-driven gene expression via binding to GREs or interfering with AP-1 and NF-κB activity, relatively little is known about the ability of GR-modulating drugs to reactivate latent HIV-1. A better understanding of the effects of GR modulators on the latent viral reservoir is of broad interest since (i) latently and productively infected cells of people living with HIV are constantly exposed to different levels of glucocorticoid hormones, (ii) corticosteroids are frequently prescribed as anti-inflammatory drugs, and (iii) the identification of novel latency-reversing agents (LRAs) may help to advance current shock-and-kill approaches in the treatment of HIV infection.

Here, we therefore tested a panel of natural and synthetic GR-modulating compounds. These comprise full agonists and antagonists, as well as partial agonists that specifically enhance the trans-repressive or trans-activating functions of GR. To identify potential cell type-specific effects, we directly compared different latently infected T lymphocytic and myeloid cell lines, as well as primary CD4+ T cells. In line with a previous study (15), we found that mifepristone suppressed LTR-mediated gene expression. More importantly, we identify AZD9567 as an LRA. This partial GR agonist reactivated latent HIV-1 in all models tested. Mutational analyses revealed that its full reactivating potential depends on AP-1 and GR binding sites in the viral LTR. Thus, we, here, identify and characterize AZD9567 as latency reversing agent, an oral GR modulator that was previously tested in phase 2a clinical trials for rheumatoid arthritis (20).

## RESULTS

### AZD9567 reactivates latent HIV-1 in lymphoid (J-Lat 10.6, ACH-2) and myeloid (U1) cell lines

Due to the presence of GREs in the HIV-1 LTR (Fig. 1C and D) and the crucial role of the GR targets AP-1 and NF-κB in HIV-1 reactivation, we hypothesized that some GR-modulating drugs may reactivate latent viral reservoirs. We therefore selected a set of steroidal and non-steroidal compounds that act as agonists or antagonists of GR (Table 1). This comprised the natural, agonistic hormones cortisol and progesterone, the clinically approved drugs dexamethasone (agonist) and mifepristone (antagonist), as well as the GR modulators dagrocorat, AZD2906, and AZD9567 that preferentially enhance the trans-repressive or trans-activating activity of GRs.

**TABLE 1:** GR modulators used in the present study^*a*^

GR-modulating compound	Mode of action	Steroidal?	Solvent used in this study	Previously described effects on HIV-1 latency
Cortisol/hydrocortisone	Full GR agonist, weak PR agonist	Yes	Water	
Progesterone	Full PR agonist, weak GR agonist	Yes	Ethanol	
Dexamethasone	Full GR agonist	Yes	Methanol	Silences HIV-1 in microglial cells (14)
Mifepristone/RU-486	Competitive PR and (to a lesser extent) GR and AR antagonist	Yes	Ethanol	Suppresses Vpr-mediated reactivation of latent HIV-1 in U1 and OM-10.1 cells (15)
Dagrocorat/PF-251802	Selective monomeric GR agonist and modulator (SEMoGRAM), enhancing trans-repression	No	DMSO	
AZD2906	Selective dimerizing GR agonist and modulator (SEDiGRAM), enhancing trans-activation	No	DMSO	
AZD9567/Mizacorat	Partial agonist enhancing trans-repression	No	DMSO	

^*a*^ GR, glucocorticoid receptor; PR, progesterone receptor; AR, androgen receptor; DMSO, dimethyl sulfoxide.

To investigate their latency reversing potential, we took advantage of three well-established models of HIV-1 latency: (i) J-Lat 10.6 cells, a T cell line latently infected with HIV-1 harboring the gene encoding green fluorescent protein (GFP) in place of nef (21), (ii) ACH-2 cells, a T cell line harboring an integrated copy of HIV-1 lymphadenopathy-associated virus (LAV) (22), and (iii) monocytic U1 cells, latently infected with HIV-1 NY5 (23). Importantly, all three cell lines showed GR protein levels similar to those in primary CD4+ T cells (Fig. 1E). Since ligand concentration determines whether GRs exert trans-repressive and/or trans-activating activities (17), we titrated all compounds over several orders of magnitude. The respective solvents (methanol, water, ethanol, or dimethyl sulfoxide (DMSO), Table 1) were included as negative controls. Cells were harvested 48 h and 96 h post stimulation, and viral reactivation was determined by quantifying the percentage of GFP-positive (J-Lat 10.6) or p24-positive (ACH-2, U1) cells via flow cytometry.

**Fig 2:**
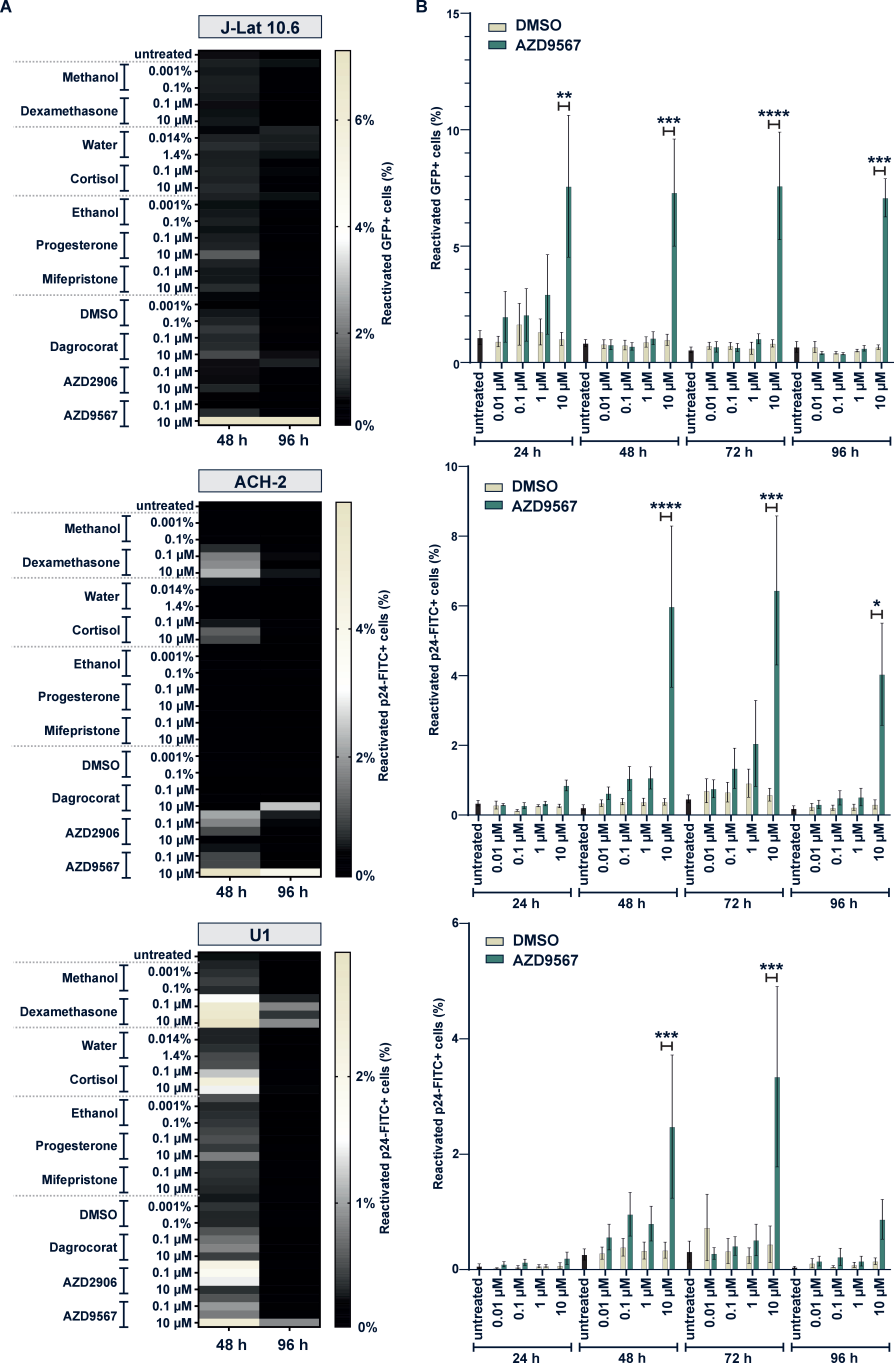
AZD9567 reactivates latent HIV-1 in lymphoid and myeloid cell lines. (**A**) J-Lat 10.6, ACH-2, and U1 cells were stimulated with increasing amounts of the indicated GR modulators (0.01 µM–10 µM) or their respective solvents (methanol, water, ethanol, or DMSO). Forty-eight hours or 96 h later, cells were fixed and the percentage of reactivated cells was determined by flow cytometry. Mean values of three to five biological replicates are shown. (**B**) J-Lat 10.6, ACH-2, and U1 cells were stimulated with increasing amounts of AZD9567 (0.01 µM–10 µM) or DMSO (0.0001%–0.1%) for 24 h, 48 h, 72 h, and 96 h and analyzed as described for (**A**). Data are shown as mean ± SEM of at least three biological replicates (*n* = 3–5). Mixed-effect analysis followed by Bonferroni’s multiple comparison test was used to compare cells treated with AZD9567 and cells treated with DMSO (*P < 0.01, **P < 0.01, ***P < 0.001, ****P < 0.0001).

The GR agonists dexamethasone and cortisol induced HIV-1 reactivation in U1 and (to a lesser extent) in ACH-2, but not in J-Lat 10.6 cells (Fig. 2A). Progesterone, a weak GR agonist, and the GR antagonist mifepristone showed no marked reactivation in any of the cell lines tested. AZD9567 was the only compound that consistently reactivated HIV-1 in all cell lines tested (Fig. 2A). Of note, AZD9567 reactivated HIV-1 only in a subfraction of the cells. However, this was also the case for the established LRAs bryostatin-1, suberoylanilide hydroxamic acid (SAHA, vorinostat), and phorbol 12-myristate 13-acetate (PMA) (Fig. S1). Furthermore, kinetics experiments with AZD9567 revealed that it reactivates HIV-1 from J-Lat 10.6 cells already within the first 24 h, while maximum reactivation of ACH-2 and U1 cells is reached between 48 h and 72 h post stimulation (Fig. 2B). Importantly, the concentrations tested (0.01 µM–10 µM) cover the serum concentrations that were reached upon oral administration in rats (2.00 µM) (24) or humans (4.45 µM) (20). Notably, AZD9567 treatment did not affect the viability of the cell lines at any time points or concentrations tested (Fig. S2). Together, these findings identify the non-steroidal, partial GR agonist AZD9567 as a drug that is able to reactivate latent HIV-1 in both lymphoid and myeloid cells.

### AZD9567 reactivates HIV-1 transcription in primary CD4+ T cells

**Fig 3:**
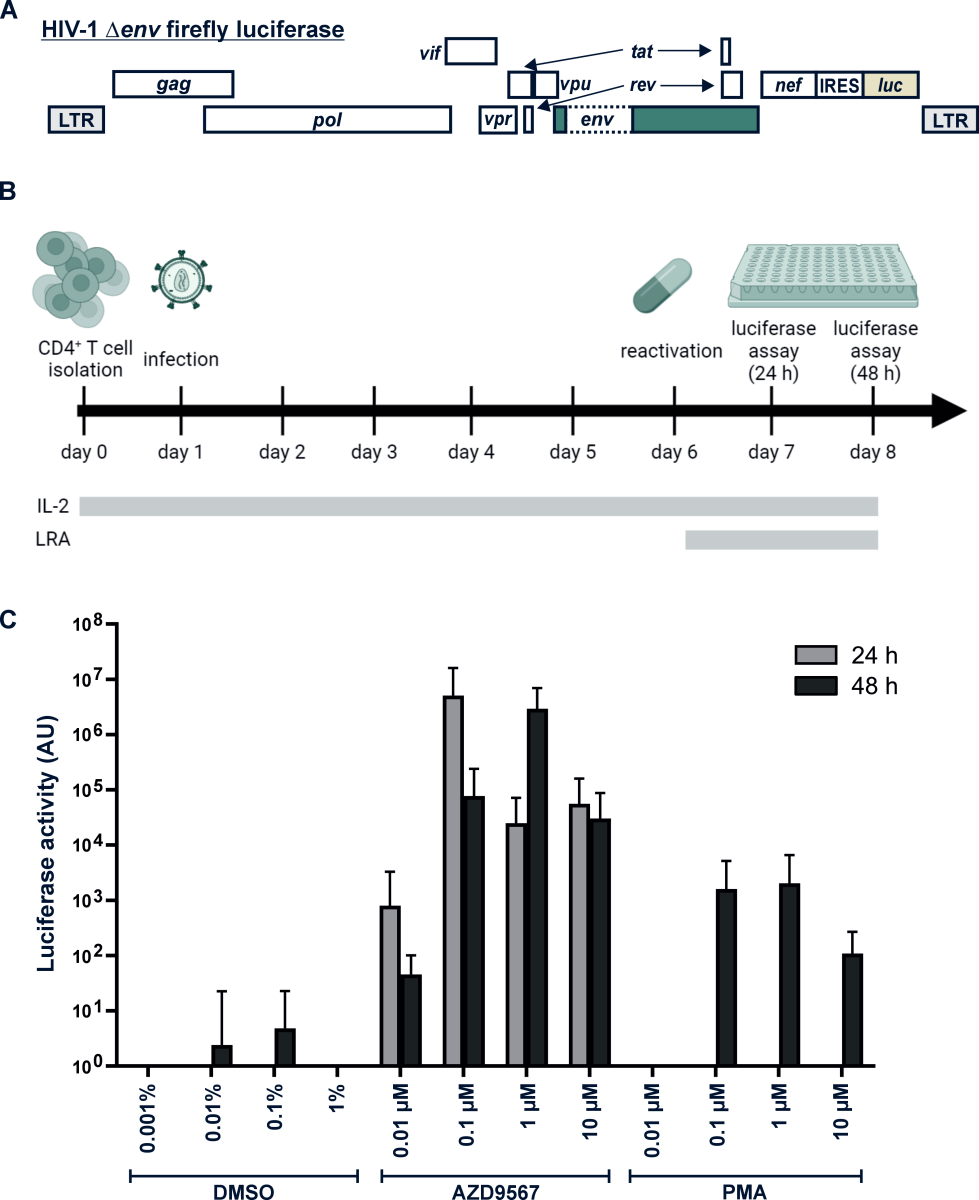
AZD9567 reactivates latent HIV-1 in primary CD4+ T cells. (**A**) HIV-1 reporter virus expressing firefly luciferase via an internal ribosome entry site (IRES) and lacking an intact *env* gene. (**B**) Experimental setup: CD4+ T cells from healthy donors were infected with the reporter virus shown in (**A**), in the presence of interleukin 2 (IL-2), but without prior activation. Five days later, cells were treated with increasing amounts of PMA, AZD9567, or DMSO. Luciferase activity was determined 24 h and 48 later. (**C**) Cells were infected, stimulated, and analyzed as described in (**B**). Mean values ± SD of five independent donors, analyzed in technical triplicates, are shown.

To examine the ability of AZD9567 to reactivate HIV-1 transcription in primary cells, we took advantage of a CD4+ T cell model based on a method developed by Lassen and colleagues (25). Briefly, CD4+ T cells from healthy donors (non-activated) were infected with an env-deficient, VSV-G pseudotyped HIV-1 firefly luciferase reporter virus (Fig. 3A). Five days post infection, no residual firefly luciferase activity was detectable, and cells were treated with increasing concentrations of AZD9567 to reactivate latent proviruses (Fig. 3B). PMA and DMSO served as positive and negative controls, respectively. Luciferase activity was determined 24 h and 48 h post stimulation. As expected, PMA stimulation effectively reactivated HIV-1 transcription, while DMSO had no or only minor effects (Fig. 3C). Notably, AZD9567 increased luciferase activity 2 to 3 orders of magnitude more efficiently than PMA. In contrast to the cell lines tested above, AZD9567 triggered luciferase expression even at the lowest concentration tested (0.01 µM), demonstrating that the GR modulator can also induce viral transcription in primary cells (Fig. 3C).

### AZD9567 activates the HIV-1 LTR promoter

**Fig 4:**
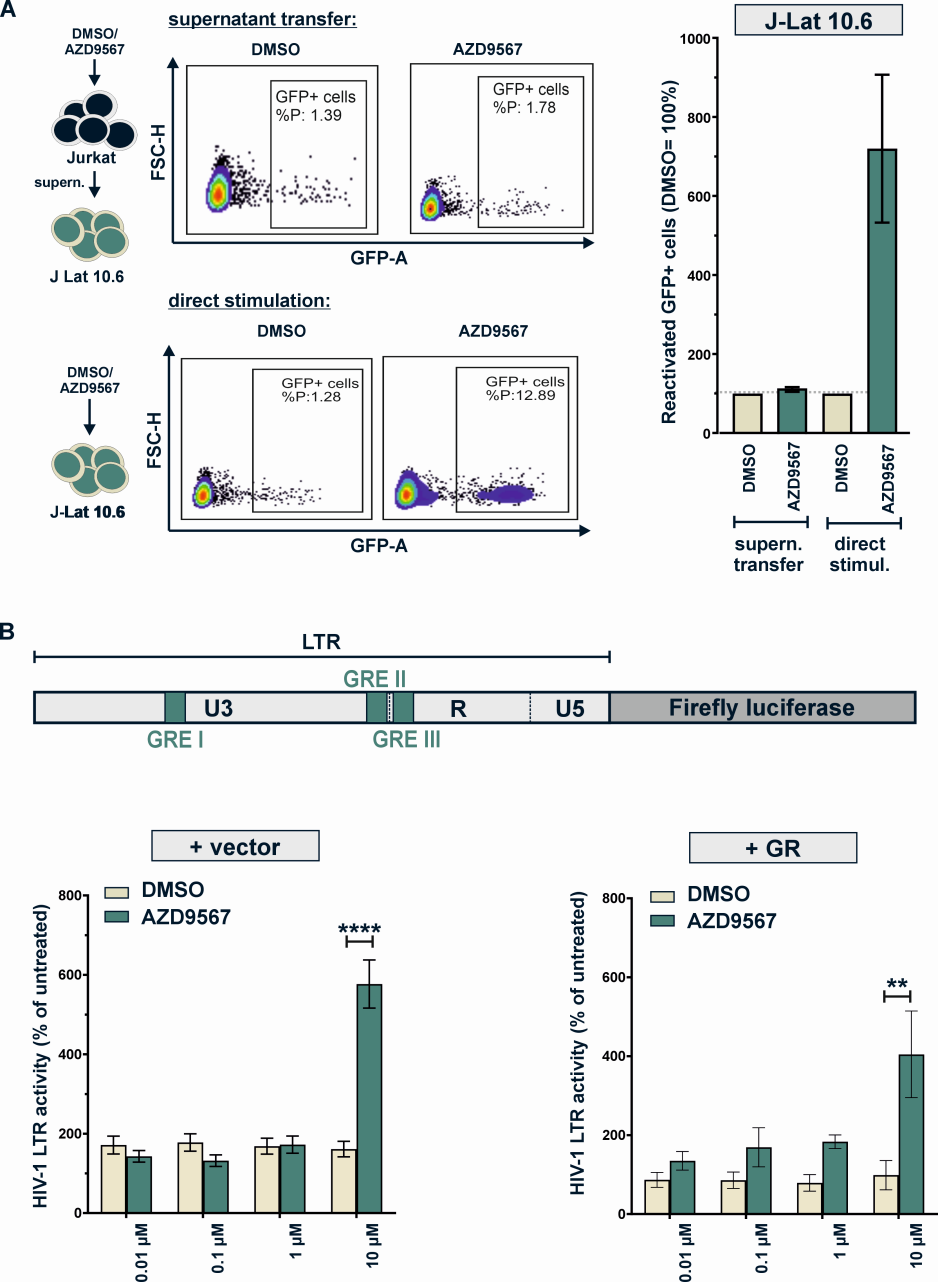
Mechanisms of AZD9567-mediated HIV-1 reactivation. (**A**) Jurkat cells (clone E6.1) were stimulated with AZD9567 (10 µM) or DMSO (0.1%). Six hours later, the culture medium was removed and transferred to J-Lat 10.6 cells. In parallel, some J-Lat 10.6 cells were directly stimulated with 10 µM AZD9567. After 48 h, cells were fixed and percentage of GFP-positive cells was determined using flow cytometry to monitor reactivation. Exemplary primary data of one out of three biological replicates are shown in the center. Mean values ± SEM of three independent biological replicates are shown on the right. (**B**) The effect of AZD9567 on HIV-1 LTR activity was analyzed using a luciferase reporter construct (top). HEK293T cells were co‐transfected with the reporter plasmid expressing firefly luciferase under the control of the HIV-1 LTR promoter and a construct expressing *Gaussia* luciferase under the control of a minimal promoter. Cells were additionally co-transfected with an empty vector control (left) or a GR expression plasmid (right). Six hours post‐transfection, cells were stimulated with the indicated amounts of AZD9567 (0.01 µM–10 µM) or DMSO (0.0001%–0.1%). Firefly luciferase activity was determined and normalized to *Gaussia* luciferase activity. Mean values ± SEM of biological replicates (*n* = 3–16) are shown and were analyzed by two‐way analysis of variance with Bonferroni’s multiple comparison test (**P < 0.01, ****P < 0.0001).

We hypothesized that AZD9567 may reactivate HIV-1 either by directly modulating LTR activity or indirectly, via altering the expression and release of LTR activating cytokines. To test the latter hypothesis, we stimulated parental Jurkat cells with AZD9567 or DMSO. After 6 h, cells were washed and incubated for another 18 h, before cell culture supernatants were transferred to J-Lat 10.6 cells (Fig. 4A). While direct treatment of J-Lat 10.6 cells with AZD9567 reactivated latent HIV-1, the supernatants of AZD9567-treated Jurkat cells showed no such activity. This strongly suggests that a soluble factor is not the main determinant of AZD9567-mediated HIV-1 reactivation.

**Fig 5:**
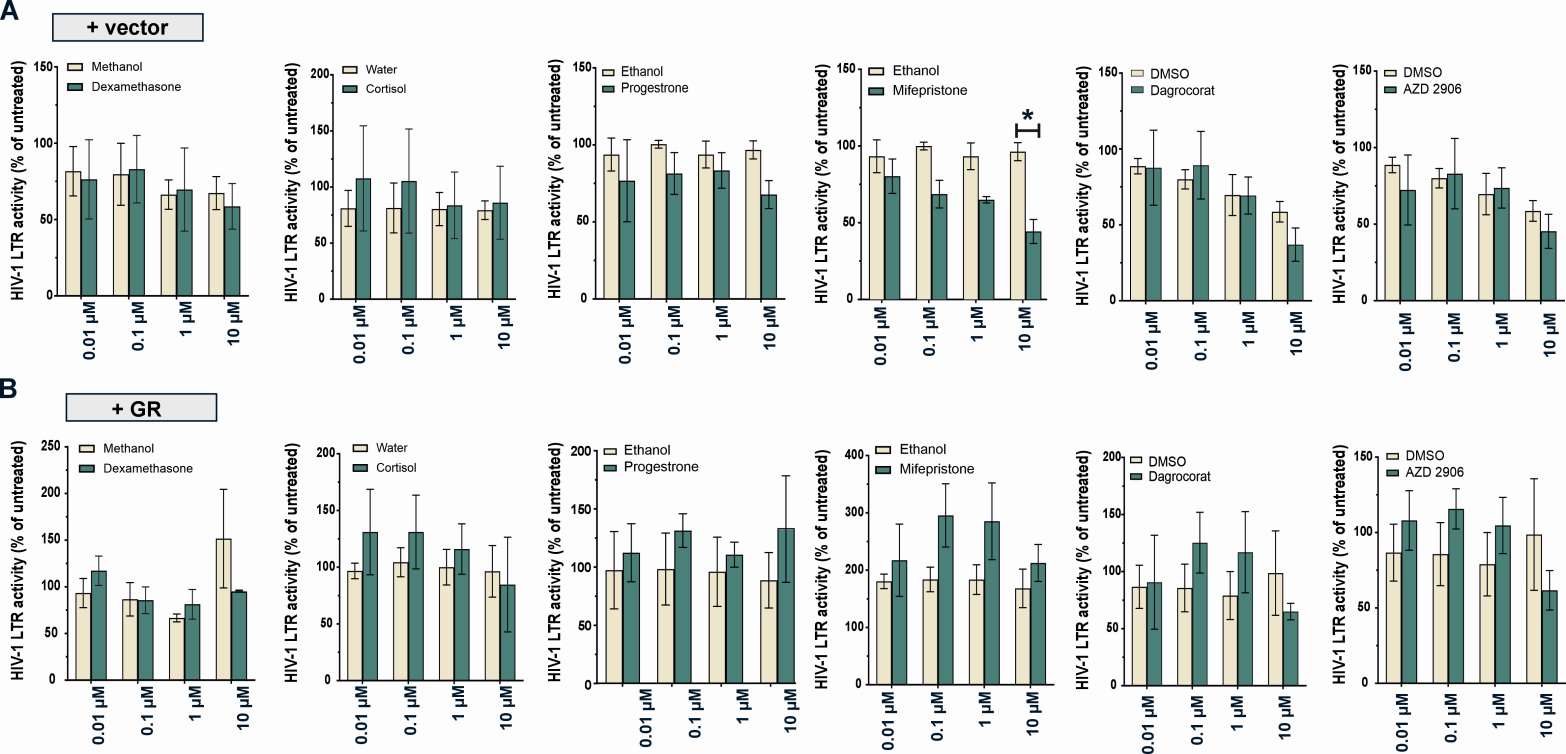
Effects of GR modulators on the activity of the HIV-1 LTR promoter. HEK293T cells were transfected with an HIV-1 LTR firefly reporter plasmid (**A**) alone or (**B**) together with a GR expression plasmid. Cells were additionally co-transfected with a *Gaussia* luciferase reporter for normalization. Six hours post‐transfection, cell culture medium was removed and fresh medium containing GR modulators (0.01 µM–10 µM) or their respective solvents (0.0001%–0.1%) were added. One day post‐transfection, firefly luciferase activity was determined and normalized to *Gaussia* luciferase activity. Mean values ± SEM of three biological replicates (*n* = 3) are shown and were analyzed by two‐way analysis of variance with Bonferroni’s multiple comparison test (*P < 0.05).

We therefore decided to investigate the direct effects of AZD9567 on the viral LTR using a firefly luciferase-based reporter assay in transfected HEK293T cells (Fig. 4B, top). While low concentrations showed no significant effects, 10 µM AZD9567 enhanced HIV-1 LTR activity (Fig. 4B, bottom left). None of the remaining compounds showed a similar enhancing effect (Fig. 5). However, 10 µM of the GR antagonist mifepristone significantly reduced basal LTR activity by about 50%, in the absence of GR over-expression (Fig. 5A). Since HEK293T cells express relatively low levels of GR (Fig. 1E), we repeated the experiment in the presence of a GR expression plasmid (Fig. 4B, bottom right). Again, AZD9567 significantly increased luciferase activity at a concentration of 10 µM, demonstrating that AZD9567 is able to enhance the activity of the viral LTR promoter.

### AZD9567-mediated LTR activation partially depends on GRE I–III

**Fig 6:**
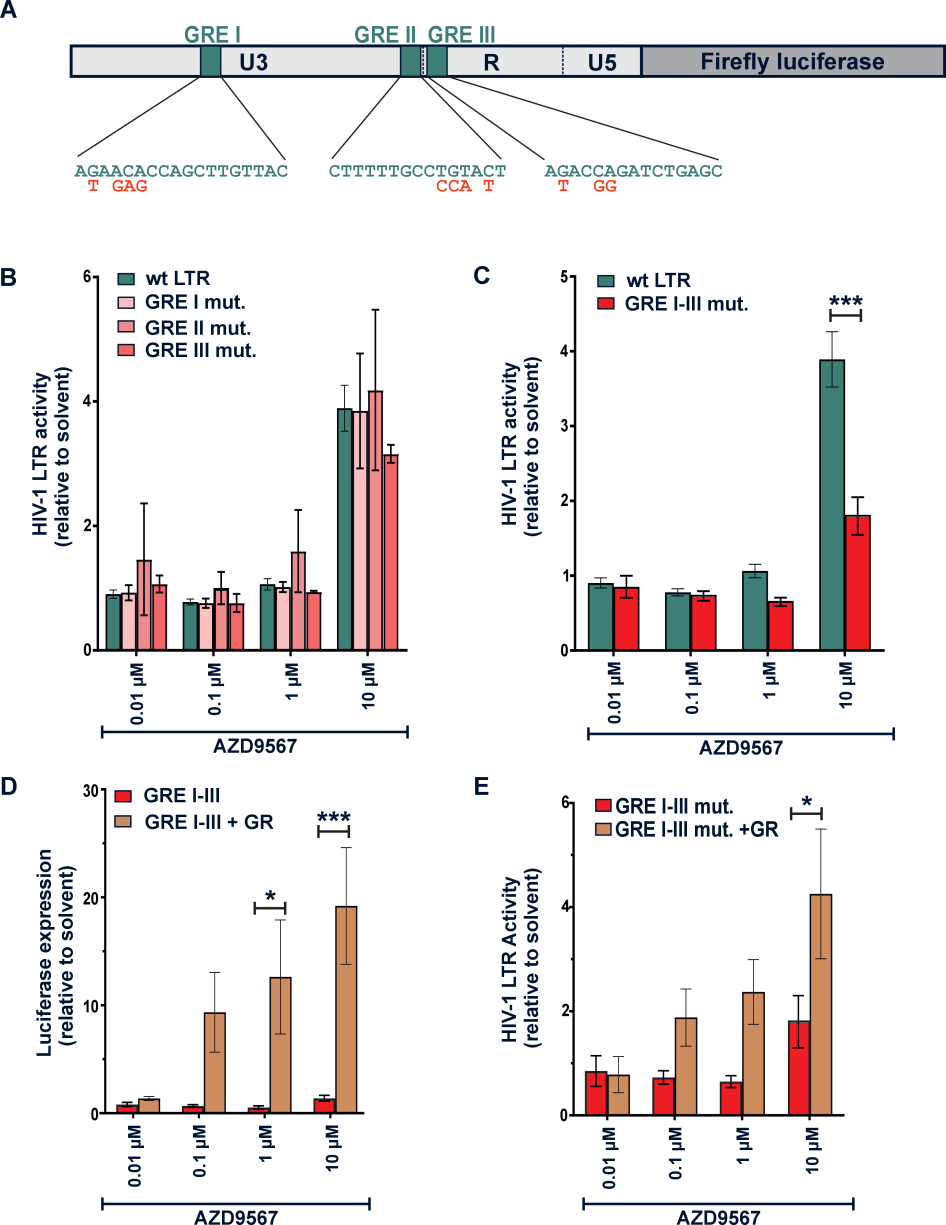
Disruption of GRE I–III partially abrogates AZD9567-mediated HIV-1 LTR activation. (**A**) Cartoon illustrating the localization of GRE I–III in the HIV-1 LTR. Mutations disrupting the GREs are shown in red. (**B**) HEK293T cells were co‐transfected with a reporter plasmid expressing firefly luciferase gene under the control of the wild-type LTR or a mutated LTR with disrupted GRE I, II, or III. Cells were co-transfected with a construct expressing *Gaussia* luciferase under the control of a minimal promoter for normalization. Six hours post‐transfection, cells were stimulated with AZD9567 (0.0.1 µM–10 µM) or DMSO (0.0001%–0.1%). Thirty hours post-transfection, firefly luciferase activity was determined and normalized to *Gaussia* luciferase activity. Mean values ± SEM of biological replicates (*n* = 3–16) are shown. (**C**) HEK293T cells were co-transfected with a firefly luciferase reporter plasmid harboring either the wild-type LTR or a mutant LTR, in which GRE I–III were disrupted simultaneously. Cells were co-transfected with a construct expressing *Gaussia* luciferase under the control of a minimal promoter for normalization and analyzed essentially as described in (**B**). Mean values ± SEM of at least four biological replicates (*n* = 4–16) are shown. (**D**) HEK293T cells were co‐transfected, stimulated, and analyzed as in (**C**). Half of the samples were additionally co-transfected with an expression plasmid for GR. Mean values ± SEM of four independent biological replicates are shown. (**E**) HEK293T cells were co-transfected with a reporter plasmid containing the GRE I–III sequences of the HIV-1 LTR upstream of a minimal promoter, the *Gaussia* luciferase reporter for normalization, and a GR expression plasmid or the respective vector control. Cells were stimulated with increasing doses of AZD9567 and analyzed as described in (**B–D**). Data are shown as mean ± SEM (*n* = 3–4). Data were analyzed by two‐way analysis of variance with Bonferroni’s multiple comparison test (*P < 0.05, ***P < 0.001).

Due to the different modes of GR action (Fig. 1A), we hypothesized that AZD9567 may activate the viral LTR by directly enhancing GR binding to one or more of the GREs and/or by interfering with the activity of other transcription factors, such as NF-κB and AP-1. Individual mutation of one of the three GREs did not abrogate responsiveness of the viral LTR to AZD9567 (Fig. 6A and B). However, simultaneous mutation of GRE I–III partially abrogated LTR activation in the presence of AZD9567 (Fig. 6C). In line with this, a luciferase reporter harboring GRE I–III of the viral LTR was responsive to AZD9567, particularly upon over-expression of GR (Fig. 6D). These findings demonstrate that at least two of the three LTR GREs are responsive to AZD9567 stimulation. Notably, however, residual responsiveness of the LTR ΔGRE I–III triple mutant (Fig. 6C) suggested that GRE I–III-independent mechanisms are also involved. In line with this, responsiveness of the triple mutant to AZD9567 could be further enhanced by over-expression of GR (Fig. 6E).

### Efficient AZD9567-mediated HIV-1 reactivation depends on an AP-1 binding site in the LTR

**Fig 7:**
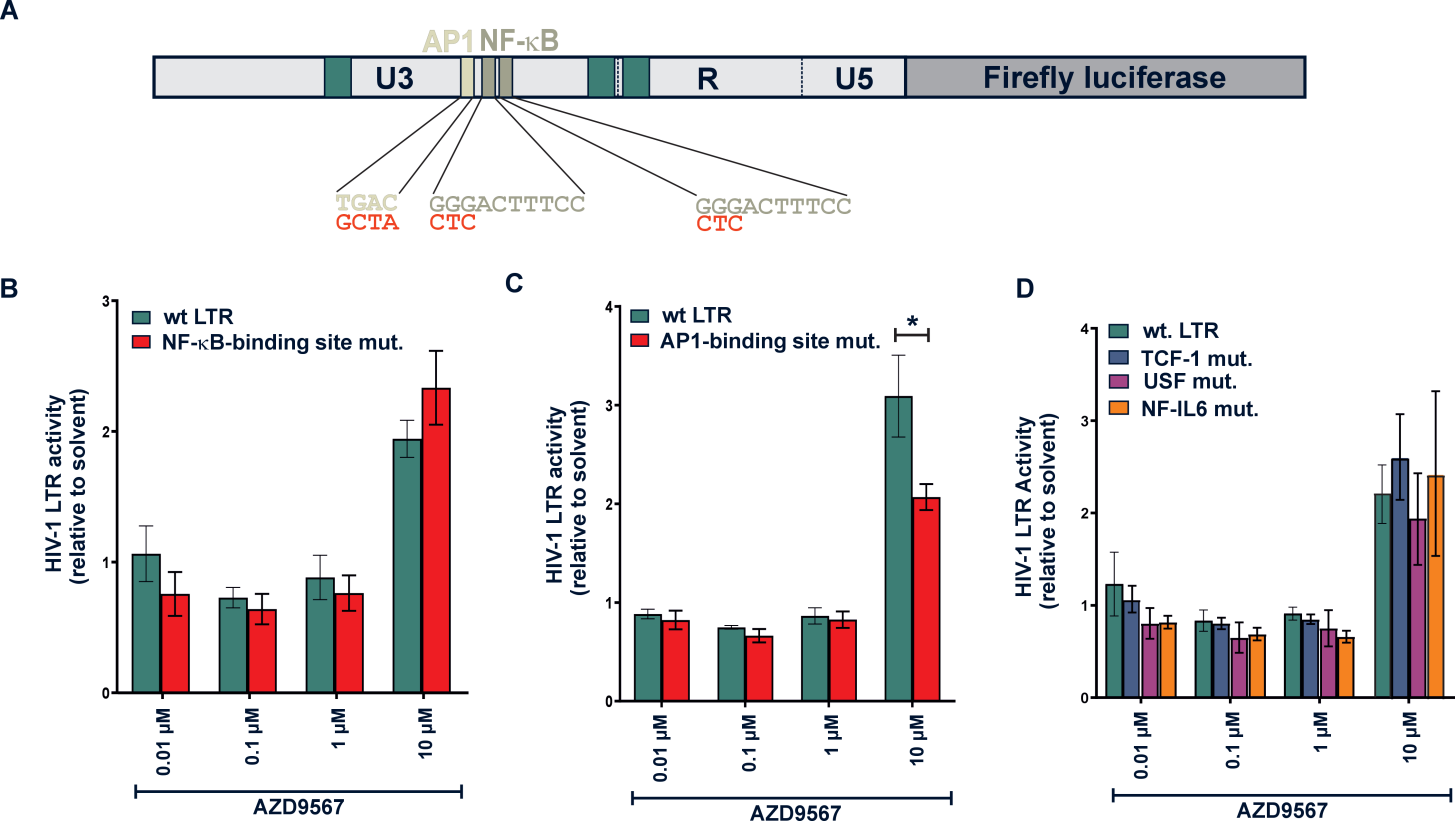
Efficient AZD9567-mediated HIV-1 reactivation depends on an AP-1 binding site in the LTR. (**A**) Schematic representation of the HIV-1 LTR reporter construct where the two NF-κB binding sites or an adjacent AP-1 binding site was disrupted. Mutated residues are highlighted in red. (**B, C**) HEK293T cells were co‐transfected with a reporter plasmid harboring either the wild-type LTR or a mutant with (**B**) disrupted NF-ᴋB binding sites or (**C**) a disrupted AP-1 binding site, and a construct expressing *Gaussia* luciferase under the control of a minimal promoter. Six hours post‐transfection, cells were treated with AZD9567 (0.0.1 µM–10 µM) or DMSO (0.0001%–0.1%). Thirty hours post-transfection, firefly luciferase activity was determined and normalized to *Gaussia* luciferase activity. Data are shown as mean ± SEM of three biological replicates (*n* = 3) and were analyzed by two‐way analysis of variance with Bonferroni’s multiple comparison test (*P < 0.05). (**D**) HEK293T cells were co-transfected with firefly luciferase LTR reporter constructs harboring mutation in the indicated transcription factor binding sites. Mean values ± SEM from three independent experiments each performed in triplicate are shown.

Since NF-κB and AP-1 are known targets of GR (26), we also mutated the two viral NF-κB binding sites or an adjacent AP-1 binding site (Fig. 7A). While mutation of the NF-κB binding sites had no significant effect (Fig. 7B), loss of the AP-1 site partially reduced AZD9567 responsiveness (Fig. 7C). Notably, mutations in the TCF-1, USF, or NF-IL6 binding sites did not affect AZD9567 responsiveness of the HIV-1 LTR either (Fig. 7D). Together, these findings demonstrate that not only GREs, but also an AP-1 binding site determines the activation of the viral LTR in the presence of the GR agonist AZD9567.

## DISCUSSION

Antiretroviral drugs can efficiently suppress ongoing HIV-1 replication. However, treatment interruption usually results in a rapid increase of viral loads due to the persistence of latently infected cells. To overcome this hurdle, current research focuses on improving therapeutic approaches that target the latent viral reservoir. Many of them can be categorized into either “block-and-lock” or “shock-and-kill” approaches. Block-and-lock strategies aim at achieving a complete silencing of integrated proviruses. In contrast, shock-and-kill approaches involve a two-step process in which latently infected cells are reactivated by LRAs before they are killed by effectors of the host immune response or synthetic drugs. Well-characterized LRAs include histone deacetylase inhibitors or histone methyltransferase inhibitors that target the integrated provirus epigenetically (27, 28). Other LRAs reactivate latent proviruses by modulating the activity of cellular transcription factors. For example, protein kinase C (PKC) agonists and SMAC mimetics have been shown to reactivate HIV-1 by activating NF-κB (29–31). More recently, the transcription factor AP-1/c-Fos was shown to synergize with NF-κB in LRA-mediated HIV-1 reactivation (10, 32). Since GRs are known to interfere with both, NF-κB- and AP-1-mediated transcriptions (33), and can directly bind to the viral LTR promoter (34), we hypothesized that some GR-modulating compounds may also act as LRAs.

By screening a set of natural glucocorticoid hormones and synthetic GR modulators, here, we identified AZD9567 as a latency reversing agent. While many LRAs act in a cell type-dependent manner (35), AZD9567 reactivated HIV-1 in both T cells and monocytes (Fig. 2 and 3). AZD9567 is a non-steroidal oral GR modulator that has been shown to be safe and well-tolerated in a phase 2a clinical trial for the treatment of rheumatoid arthritis (20). It is a partial GR agonist that was developed from a full GR agonist to reduce side effects while maintaining potent anti-inflammatory activity (24). Full GR agonists such as dexamethasone induce both, trans-repressive and trans-activating effects of GRs (Fig. 1A). While full GR agonists enable suppression of pro-inflammatory immune responses (e.g., TNF-α expression), they may also result in unwanted adverse effects such as hyperglycemia, skin atrophy, or bone resorption. To separate beneficial from detrimental effects and to induce only a subset of GR-mediated pathways, selective glucocorticoid receptor modulators are developed. AZD9567, for example, exerts full trans-repressive but only partial trans-activating effects (24). In line with the induction of several GR-mediated pathways, we found that AZD9567 activates HIV-1 LTR-driven gene expression by at least two different mechanisms: the first mechanism involves the presence of GREs in the viral LTR promoter. To date, three different LTR GREs (GRE I–III) have been described (Fig. 1B and C) (9). Although they do not perfectly match the palindromic consensus binding site of GR, they are conserved among different subtypes of HIV-1 (Fig. 1D), and their combined mutation partially abrogated responsiveness of the LTR to AZD9567 (Fig. 6C). Notably, however, individual disruption of single GREs had no significant effect suggesting that at least two of the three GREs mediate AZD9567 responsiveness, most likely via direct binding of GR. The observation that the LTR GRE I–III triple mutant is still partially responsive to AZD9567 (Fig. 6C and E) raised two possibilities: (i) the presence of additional GREs in the viral LTR and/or (ii) modulation of other transcription factors by AZD9567. In support of the second hypothesis, mutation of an AP-1 binding site also partially abrogated responsiveness of the viral LTR to AZD9567 stimulation (Fig. 7C). While AP-1 is a known target of GR, this finding came as a surprise since GR is generally thought to inhibit rather than activate AP-1-mediated gene expression (33). Thus, GR-mediated modulation of other transcription factors may be more complex than initially thought. Notably, AP-1 itself also seems to play a dual role in HIV-1 reactivation. On the one hand, AP-1 has been shown to contribute latency reversal upon T cell stimulation (36, 37). On the other hand, AP-1 also seems to promote establishment of viral latency at the of infection (32).

In principle, full GR agonists are expected to also exert the activities of partial agonists such as AZD9567. However, dexamethasone and cortisol only reactivated latent HIV-1 in U1 cells, but not in J-Lat 10.6 cells and only poorly in ACH-2 cells. This may be explained by differences in GR binding kinetics and/or opposing effects of trans-activating and trans-repressing activities on HIV-1 latency. With 1 µM–10 µM, the concentrations of AZD9567 required to reactivate latent HIV-1 are relatively high, although similar serum concentration could be reached upon oral administration in rats and humans (20, 24). It is tempting to speculate that the generation of (more) selective GR modulators will help to further increase the reactivating potential of this class of drugs. Importantly, the current study is limited to experiments *in vitro*, i.e., latently infected cell lines and *ex vivo* infected primary CD4+ T cells. While these latency models provide a valuable starting point, it remains to be determined whether AZD9567 or similar compounds are also able to reactivate dormant proviruses in primary cells *in vivo*. Another limitation is the use of only a few (cell culture-adapted) HIV-1 clones. Since different subtypes of HIV-1 differ in the number and sequence of GREs and other transcription factor binding sites, it will be important to test responsiveness of additional HIV-1 clones, preferentially representing primary isolates of the virus.

Apart from optimizing current shock-and-kill approaches, a better characterization of GR modulators in the context of HIV-1 latency will also improve our understanding of the effects of natural glucocorticoid hormones on the course of HIV-1 infection. In this context, it will also be important to consider gender-specific differences since women and men react differently to glucocorticoid stimulation (38). Finally, potential effects on latent HIV-1 should be considered when treating infected individuals with (novel) GR-modulating drugs.

## MATERIALS AND METHODS

### Sequence logo plots and alignments

Weblogos of (putative) GREs in the HIV-1 genome were generated using AnalyzeAlign (https://www.hiv.lanl.gov/content/sequence/ANALYZEALIGN/analyze_align.html). The following parameters were used: LANL database alignment type: “Subtype reference”; Organism: “HIV-1/SIVcpz”; Sequence type: “nucleotide”. Alignments were generated using MultAlin (39).

### Cell culture

All cells were cultured at 37°C, 90% relative humidity, and 5% CO_2_. HEK293T cells were obtained *via* the ATCC and maintained in Dulbecco’s modified Eagle medium supplemented with 10% heat-inactivated fetal calf serum (FCS), L-glutamine (2 mM), streptomycin (100 mg/mL), and penicillin (100 U/mL). They were isolated from a female fetus (40). HEK293T cells were transfected using the calcium phosphate method. ACH-2 cells are a derivative of CEM A3.01 cells, which in turn were derived from T cell acute lymphoblastic leukemia cells of a 3-year-old Caucasian girl (22). They harbor one integrated proviral copy of latent HIV-1 LAV (22). U1 cells are a derivative of U937 cells, which represent pro-monocytes obtained from a pleural effusion of a 2-year-old Caucasian male with diffuse histiocytic lymphoma (23). They harbor one integrated proviral copy of latent HIV-1 (23). J-Lat 10.6 cells are derived from Jurkat cells which were derived from T cell acute lymphoblastic leukemia cells of a 14-year-old Caucasian boy (21). They harbor a full-length integrated HIV-1 genome that expresses GFP upon activation (21). The genome generates non-infectious virions due to a frameshift in the *env* gene. All latently infected cell lines were kindly provided by Frank Kirchhoff and originally obtained through the NIH HIV AIDS reagent program (ACH-2: cat# ARP-349; U1: cat# ARP-165; J-Lat 10.6: cat# ARP-9849) and cultured according to the provider’s recommendations. CD4+ T cells were negatively isolated from lymphocyte concentrates from 500 mL whole blood using the RosetteSep Human CD4+ T Cell Enrichment Cocktail (Stem Cell Technologies) according to the manufacturer’s instructions. Primary cells were cultured in RPMI-1640 medium containing 10% FCS, 2 mM glutamine, 100 µg/mL streptomycin, 100 units/mL penicillin, and 10 ng/mL interleukin 2 (IL-2). Before analysis, cells were stimulated for 2 to 3 days with 1 µg/mL phytohemagglutinin.

### Expression plasmids

The glucocorticoid receptor gene was PCR amplified using human cDNA as a template and inserted into a pCG expression vector co-expressing blue fluorescent protein (BFP) *via* an IRES using XbaI and MluI restriction sites.

### Western blot

To determine expression of GR, cells were washed in phosphate-buffered saline (PBS), lysed in co-immunoprecipitation (co-IP) lysis buffer (150 mM NaCl, 50 mM HEPES, 5 mM EDTA, 0.1% NP40, 500 mM Na_3_VO_4_, and 500 mM NaF, pH 7.5) containing protease and phosphatase inhibitors at 4°C for 20 min. The lysates were then cleared by centrifugation at 20,000* g* for 20 min at 4°C, mixed with protein sample loading buffer (LI-COR, cat# 928-40004), supplemented with 10% β-mercaptoethanol, and heated at 95°C for 5 min. Proteins were separated on NuPAGE 4%–12% Bis–Tris Gels (Thermo Fischer Scientific, cat# NP0323BOX), blotted onto Immobilon-FL PVDF membranes (Merck Milipore, cat# IPFL00010), and stained using primary antibodies directed against GR (cell signaling, cat# 12,041s) or GAPDH (Biolegend, cat# 607902) and infrared dye-labeled secondary antibodies (LI-COR IRDye). All washing and blocking steps were performed using PBS containing 0.2% Tween 20 and 1%–5% milk. Proteins were detected using a LI-COR Odyssey Fc scanner.

### Stimulation of HIV-1 reporter cell lines and flow cytometry

ACH-2, J-Lat 10.6, or U1 cells were seeded in round bottom 96-well plates (0.15–0.2 × 10^6^ cells/well). On the same day, cells were stimulated with the indicated amount of dexamethasone (Sigma-Aldrich, cat# D1756) dissolved in methanol, cortisol (Sigma-Aldrich, cat# H4001) dissolved in water, progesterone (Sigma-Aldrich, cat# p8783) or mifepristone (Sigma-Aldrich, cat# 475838) dissolved in ethanol, dagrocorat (Aobious, cat# AOB87471), AZD2906 (MedChemExpress, cat# HY-113854), AZD9567 (MedChemExpress, cat# HY-120012), SAHA (Stemcell, cat# 73902), PMA (Sigma-Aldrich, cat# P1585-1MG), or bryostatin-1 (R&D Systems, cat# 2383) dissolved in DMSO. The respective solvents were included as negative controls. HIV-1 reactivation was monitored using flow cytometry 24 to 96 h after treatment. To this end, J-Lat 10.6 cells were washed in PBS with 2% FCS, fixed in 2% paraformaldehyde (PFA), and the percentage of GFP-positive cells was determined using a MACS Quant VYB (Miltenyi Biotec). ACH-2 and U1 cells were washed with PBS, fixed, and permeabilized with the FIX & PERM kit (Nordic-MUbio, #GAS-002-1) according to the manufacturer’s instructions before they were stained for HIV-1 capsid protein using an anti-p24 antibody conjugated with fluorescein isothiocyanate (FITC) (Beckman Coulter, cat# 6604665). A MACS Quant VYB (Miltenyi Biotec) was used to determine the percentage of p24-positive cells.

### *Ex vivo* reactivation model in CD4+ T cells

One day after isolation, primary, non-activated CD4+ T cells were infected with a VSV-G-pseudotyped *env*-defective HIV-1 reporter virus expressing firefly luciferase via an IRES (HIV-1 NL4-3 Δ*env* IRES firefly luciferase). For infection, a standard spinoculation protocol was used (1,200 × *g* for 2 h at 37°C; 10^5^ cells per well of a 96-well plate). Cells were cultured in the presence of IL-2 for 5 days, before latency reversing agents (PMA, AZD9567) or the respective solvent control (DMSO) was added in technical triplicates. Viral transcription was monitored by quantifying firefly luciferase activity 24 h and 48 h post stimulation. The signal of mock infected cells was subtracted.

### Cell viability assay

The CellTiter-Glo Luminescent Cell Viability Assay (Promega, Cat# G7571) was used to determine the effect of AZD9567 on cell viability according to the manufacturer’s instructions. Briefly, ACH-2, J-Lat 10.6, or U1 cells were seeded in 96-well U-bottom plates (0.15–0.2 × 10^6^ cells/well) and treated with increasing doses of AZD9567 (0.01, 0.1, 1, and 10 µM). The solvent controls at appropriate concentrations were included as negative controls. One hundred microliters of CellTiter-Glo Reagent was added in each well 24 to 96 h after treatment, and the cells were incubated at room temperature for 10 min. The cell lysates were then transferred to a 96-well opaque plate, and luminescence was recorded using Berthold Multimode Microplate Reader (Berthold Technologies, Georgia).

### LTR, GRE, AP-1, and NF-κB reporter plasmids

The promoter reporter vector harboring the 5′ LTR of HIV-1 NL4-3 was constructed by inserting the HIV-1 LTR sequence upstream of the firefly luciferase gene in pGL4.32 [luc2P/NF-kB-RE/Hygro] (Promega E8491) (41). Overlap extension PCR was used to generate LTR reporter vectors harboring mutated GREs or AP-1 binding site. GRE I–III were inserted into pGL4.32 [luc2P/NF-kB-RE/Hygro] (Promega E8491) upstream of the minimal promoter, *via* NheI/EcoRI. All primers used for the generation of reporter constructs are listed in Table S1. pGL3-enhancer HIV-1 M NL4-3 LTR reporter plasmids with mutated binding sites for NF-κB/NFAT-I/II, TCF-1α, USF, and NF-IL-6 were kindly provided by Frank Kirchhoff (Ulm, Germany) and have been described previously (42).

### Luciferase reporter assays

HEK293T cells were seeded in 96‐well plates at a density of 2 × 10^4^ cells/well. On the following day, cells were transfected using the calcium phosphate method with a mixture of firefly luciferase reporter constructs, along with a *Gaussia* luciferase expression plasmid for normalization (15 ng), and an empty vector to adjust total DNA amounts across all conditions to 250 ng/well. For reporter constructs harboring wild-type or mutated LTRs, 10 ng plasmid DNA per well were used. For reporter constructs harboring GRE I–III, 160 ng plasmid DNA per well were used. In some experiments, cells were additionally co-transfected with a glucocorticoid receptor expression plasmid (80 ng/well). Supernatants were removed 24 h later, and cells were lysed in 100 µL 1× Passive Lysis Buffer (Promega, cat# E194A). *Gaussia* luciferase activity in the supernatants was measured by addition of coelenterazine (PJK Biotech, cat# 102174). Firefly luciferase activity was measured in the cells using the Luciferase Assay System (Promega, cat# E1501) according to the manufacturer’s instructions. The luminescence of the negative control, in which cells were not treated with either stimulant or solvent, was used to normalize the samples and set to 100%.

### Jurkat supernatant transfer assay

Jurkat (clone E6.1) cells were seeded at a density of 1.5–2.0 × 10^5^ cells/well in a 96-well U-bottom plate and stimulated with AZD9567 (10 µM) or DMSO (0.1%) for 6 h. The plate was then centrifuged at 1,500 rpm for 5 min, and supernatant was replaced with fresh medium. Supernatants were harvested 24 h post stimulation and added to J-Lat 10.6 cells and incubated for 48 h at 37°C. All cells were cultured in RPMI 1640 with 10% fetal bovine serum (FBS), 1% penicillin-streptomycin, and maintained at 37°C, with 90% relative humidity and under 5% CO_2_. HIV-1 reactivation was monitored *via* flow cytometry, and the percentage of GFP-positive cells was determined using a MACS Quant VYB (Miltenyi Biotec).

### Statistical analysis

Statistical analyses were performed using GraphPad PRISM 9.4.1. For statistical testing, *P*-values were calculated using two-way analysis of variance or mixed model followed by Bonferroni test for comparison between multiple groups. Unless otherwise stated, data are shown as the mean of at least three biological triplicates ± SEM. Statistical parameters are specified in the figure legends.

## SUPPLEMENTAL MATERIAL

Supplemental figures jvi.01886-24-s0001.pdf Figures S1 and S2.

ASM does not own the copyrights to Supplemental Material that may be linked to, or accessed through, an article. The authors have granted ASM a non-exclusive, world-wide license to publish the Supplemental Material files. Please contact the corresponding author directly for reuse.
