# Supplementary material for: Reactivation of latent HIV-1 by the glucocorticoid receptor modulator AZD9567
Source: J Virol. 2025 Jan 16;99(2):e01886-24. doi: 10.1128/jvi.01886-24 (PMC11853017; doi:10.1128/jvi.01886-24)
Supplement: Supplemental figures — Figures S1 and S2. [file jvi.01886-24-s0001.pdf]

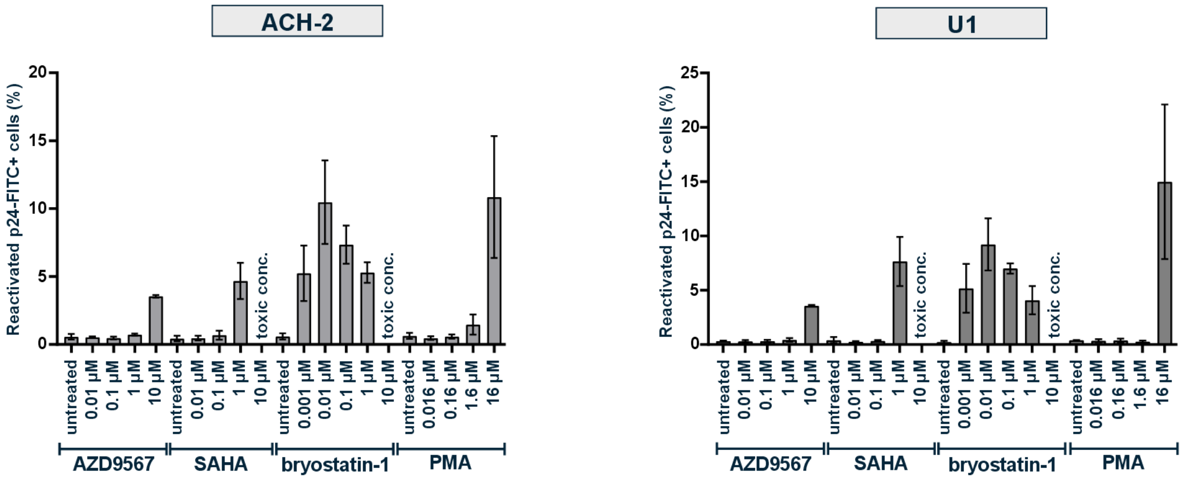

FIG S1 Comparison of the latency reversing potential of AZD956, SAHA, bryostatin-1 and PMA. ACH-2 (left) or U1 cells (right) were stimulated with increasing amounts of the indicated compounds or left untreated. 48 h later, cells were fixed and stained with p24-FITC antibody. Percentage of reactivated cells was determined by flow cytometry. Mean values of 3 biological replicates ± SD are shown.

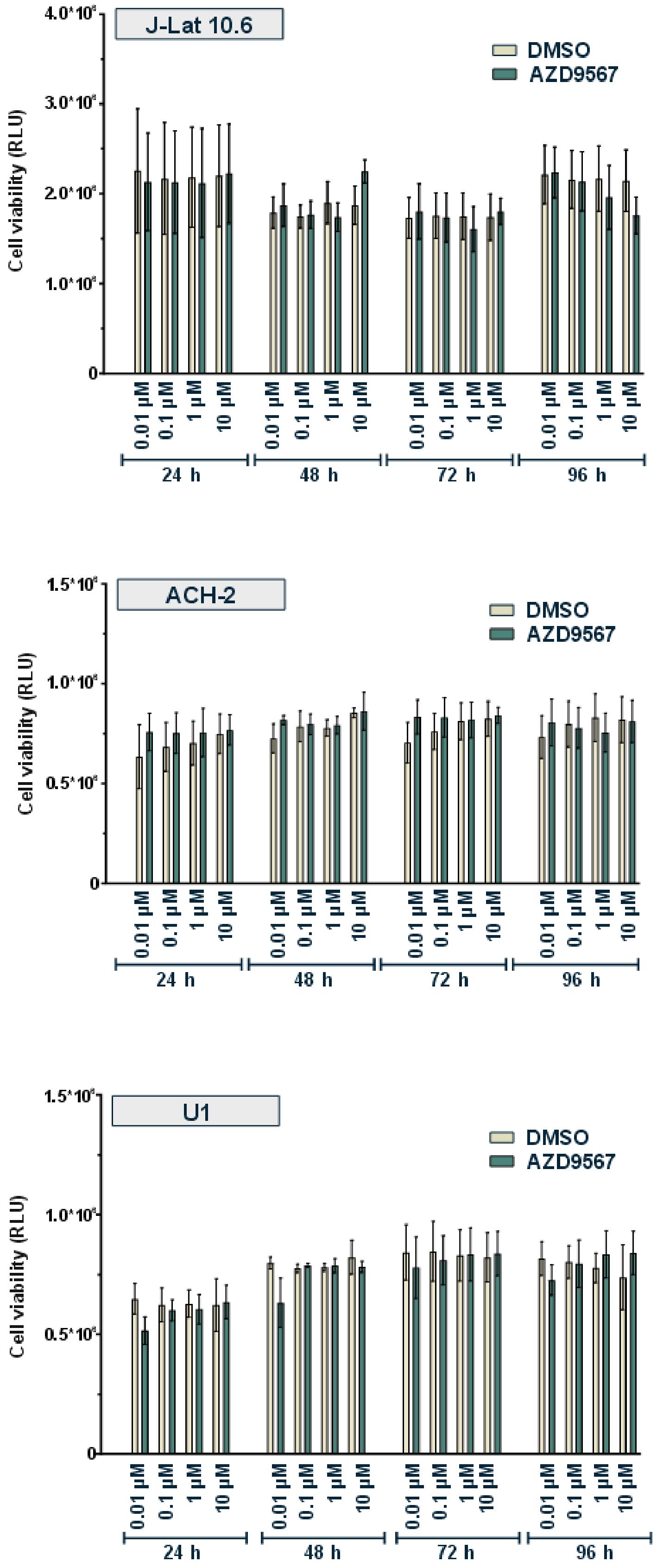

FIG S2 Viability of U1, ACH-2, and J-Lat 10.6 cell lines treated with AZD9567. Cells were treated with increasing amounts of AZD9567 (0.01 – 10 µM) or DMSO (0.0001 – 0.1%). At the indicated time points post treatment, viability was analyzed using a cell titer glow assay. Mean values ± SEM of three replicates are shown.
